# Supplementary material for: Simple models including energy and spike constraints reproduce complex activity patterns and metabolic disruptions
Source: PLoS Comput Biol. 2020 Dec 21;16(12):e1008503. doi: 10.1371/journal.pcbi.1008503 (PMC7785241; doi:10.1371/journal.pcbi.1008503)
Supplement: S1 Text — (PDF) [file pcbi.1008503.s001.pdf]

Supplementary Information

Simple models including energy and spike constraints reproduce complex activity patterns and metabolic disruptions

Tanguy Fardet & Anna Levina

Contents

A Benchmarks **2**

B Fixed points and bifurcations of the *e*LIF model **2**

B.1 Nullclines 2

B.2 Saddle-node bifurcation via $\mathbf{I_e}$ 2

B.3 General solution for the fixed points 3

C Fixed points and bifurcations of the mAdExp model **4**

D Behaviors **4**

E Parameters **5**

E.1 Neuronal parameters 6

E.2 Network simulation 8

A. Benchmarks

The runtime of the models was measured using NEST 2.20 [1] and compared with existing implementations. The neurons were parametrized to spike at 25 Hz during 60 s and compared to a baseline run of 60 s without any neuron model. Table S1 Table compares the runtime of all models mentioned in this papers, as well as conductance-based neurons.

As can be seen from S1 Table, the runtime of the models are similar to or faster than those of the AdExp and conductance-based models, while accounting for energy dynamics and displaying a larger variety of behaviors.

B. Fixed points and bifurcations of the eLIF model

B.1. Nullclines

The two nullclines of the model are given by:

$$\begin{cases} V_{Vn} &= E_0 + \dfrac{I_e}{g_L} + (E_u - E_0)\left(1 - \dfrac{\epsilon}{\epsilon_0}\right) \\ \\ V_{\epsilon n} &= E_f + (E_d - E_f)\left(1 - \dfrac{\epsilon}{\alpha\epsilon_0}\right)^3 \end{cases} \tag{S1 Eq}$$

B.2. Saddle-node bifurcation via $\mathbf{I_e}$

For a state where 3 FPs are present (see Fig 2 in the main text), the coalescence of the higher stable FP, S_+, and the unstable FP, U, occurs at a point $B = (V_B, \epsilon_B)$, when the V-nullcline touches the 3rd order polynomial, i.e. when the local slope of the tangent to the curve is equal to

$$-\frac{E_u - E_0}{\epsilon_0} = -\frac{3(E_d - E_f)}{\alpha\epsilon_0}\left(1 - \frac{\epsilon}{\alpha\epsilon_0}\right)^2 \tag{S2 Eq}$$

which leads to

$$\begin{cases} \epsilon_B &= \alpha\epsilon_0\left(1 \pm \sqrt{\frac{\alpha(E_u - E_0)}{3(E_d - E_f)}}\right) \\ V_B &= E_f \mp \frac{1}{\sqrt{E_d - E_f}}\left[\frac{\alpha}{3}(E_u - E_0)\right]^{3/2} \end{cases} \tag{S3 Eq}$$

Using also the second equation for V_B, one gets the two critical values for $I_e = \pm I_e^*$

$$\frac{I_e^*}{g_L} = (E_f - E_0) \pm \frac{1}{\sqrt{E_d - E_f}}\left[\frac{\alpha}{3}(E_u - E_0)\right]^{3/2} - (E_u - E_0)\left[1 - \alpha\left(1 \pm \sqrt{\frac{\alpha}{3}\frac{E_u - E_0}{E_d - E_f}}\right)\right] \tag{S4 Eq}$$

$$= E_f - E_0 + \alpha(E_u - E_0)\left(1 - \frac{1}{\alpha} \pm \frac{2}{3}\sqrt{\frac{\alpha(E_u - E_0)}{3(E_d - E_f)}}\right) \tag{S5 Eq}$$

**Model**	None	LIF	AdExp	*e*LIF	mAdExp	HH	HH+Ca
**Runtime (s)**	0.75	0.8	2.7	2.86 (1.79)	3.52 (2.56)	3.47	4.92

S1 Table. Runtime of various models in NEST. A "baseline" run with no neuron (None), compared to runs with one neuron of each of the mentioned models. For the new energy-based models (*e*LIF and mAdExp), two runs were performed: one using a naive implementation and another using slightly optimized implementation (numbers in parentheses). Conductance-based models are also included: a standard Hodgkin-Huxley (HH) model which can display regular spiking an depolarization block, and one with calcium and calcium-gated potassium (HH+Ca) to reproduce bursting dynamics

Which can be further simplified to give 3.

B.3. General solution for the fixed points

The FPs of the eLIF model are the intersection of the two nullclines given by S1 Eq. Writing out the equation for the FPs results in the 3rd order polynomial. From [2], we can get the general solution for the roots of this 3rd order polynomial in the case where $E_u > E_0$. Let us write it under the form $ax^3 + bx^2 + cx + d$, given $x = \epsilon/\epsilon_0$

Coefficients here are given by:

- $a = (E_d - E_f)/\alpha^3$, $b = -3(E_d - E_f)/\alpha^2$, $c = 3(E_d - E_f)/\alpha - (E_u - E_0)$, $d = E_u - E_d + I_e/g_L$
- $x_N = -b/(3a) = \alpha$, $y_N = 2b^3/27a^2 - bc/3a + d = E_u - E_f - \alpha(E_u - E_0) + I_e/g_L$
- $\delta^2 = (b^2 - 3ac)/9a^2 = \alpha^3(E_u - E_0)/\left[3(E_d - E_f)\right]$
- $h = 2a\delta^3 = 2(E_d - E_f)\left[\frac{\alpha(E_u - E_0)}{3(E_d - E_f)}\right]^{3/2}$

Note that, though δ was used for coherence with [2], it is *not* related to the δ parameter which appears in Equation 1 in the main text and is associated with the spiking cost in the neuronal model.

3 real solutions If $I_e \in [I^*_{e-}, I^*_{e+}]$, we define

$$\theta = \frac{1}{3}\arccos\left(\frac{-y_N}{h}\right)$$

and get

$$r_k \;=\; x_N + 2\delta\cos\left(\theta + \frac{2(k-1)}{3}\pi\right) \;=\; \alpha + 2\alpha\sqrt{\frac{\alpha(E_u - E_0)}{3(E_d - E_f)}}\cos\left(\theta + \frac{2(k-1)}{3}\pi\right) \quad \text{for } k \in \{1, 2, 3\}$$

which leads to

$$\epsilon_k = \epsilon_0(1 - r_k)$$

At the bifurcation points If $I_e = I^*_{e\pm}$, one recomputes δ as $-\sqrt[3]{\frac{y_N}{2a}}$ to get its correct sign.

This gives

$$r = \delta = -\left[\frac{1}{2} + \frac{\alpha(E_u - E_0)}{2(E_d - E_f)}\left(1 - \frac{2}{\alpha} \pm \frac{2}{3}\sqrt{\frac{\alpha(E_u - E_0)}{3(E_d - E_f)}}\right)\right]^{1/3}$$

Then

$$\epsilon_1 = \epsilon_0(1 + r),\ \epsilon_2 = \epsilon_0(1 - 2r)$$

Single real solution In the case where $I_e \notin [I^*_{e-}, I^*_{e+}]$ or $E_u \leq E_0$, the single real root and is obtained through Cardano's formula:

$$r = -\frac{b}{3a} + \left[-\frac{q}{2} + \sqrt{\frac{q^2}{4} + \frac{p^3}{27}}\right]^{1/3} + \left[-\frac{q}{2} - \sqrt{\frac{q^2}{4} + \frac{p^3}{27}}\right]^{1/3} \qquad \text{(S6 Eq)}$$

with $p = \frac{c}{a} - \frac{b^2}{3a^2}$, $q = 2\left(\frac{b}{3a}\right)^3 - \frac{bc}{3a^2} + \frac{d}{a}$ and $\epsilon = \epsilon_0(1 + r)$

In all cases, the associated values of V can then directly be calculated from the equation of one of the nullclines in S1 Eq.

C. Fixed points and bifurcations of the mAdExp model

Nullclines

The nullclines of the mAdExp model can be expressed in multiple ways, among which:

$$\begin{cases} \epsilon_{Vn}(V,w) &= \epsilon_0 \dfrac{E_u - V - \Delta_T \frac{\epsilon_c}{\epsilon_0} \exp\left[(V-V_{th})/\Delta_T\right] + (I_e - w)/g_L}{E_u - E_0 - \Delta_T \exp\left[(V-V_{th})/\Delta_T\right]} \\ V_{\epsilon n}(\epsilon,w) &= E_f + (E_d - E_f)\left[\left(1 - \dfrac{\epsilon}{\alpha\epsilon_0}\right)^3 - \dfrac{w}{\gamma}\right] \\ V_{wn}(\epsilon,w) &= E_0 + (E_u - E_0)\left(1 - \dfrac{\epsilon}{\epsilon_0}\right) + \dfrac{w}{a} - \dfrac{\epsilon_c}{\epsilon_c + 2\epsilon} I_{KATP} \end{cases} \tag{S7 Eq}$$

Approximation of the fixed points

In this section, we consider parameter sets where the effect of I_{KATP} is negligible. As long as the fixed points have a value of V_{FP} which is lower than $V_{th} - \Delta_T$, their value can be well approximated by replacing g_L by $(g_L + a)$ in the solutions of the *eLIF model* (see previous section), then considering:

$$w_{FP} = a(V_{FP} - E_L) + \frac{\epsilon_c}{\epsilon_c - 2\epsilon} I_{KATP} \tag{S8 Eq}$$

Numerically, on can then converge iteratively towards an improved solution, starting from this initial guess FP_0, then correcting the external current that will be used to compute FP_{i+1} by $I_{e,i+1} = I_e - w_{FP,i} + g_L \Delta_T \frac{\epsilon_{FP,i} - \epsilon_c}{\epsilon_0} \exp\left(\frac{V_{FP,i} - V_{th}}{\Delta_T}\right)$.

D. Behaviors

This section provides some additional information regarding the behaviors that can be obtained through the eLIF and mAdExp models.

S1 Fig shows how different parameters can give rise to both type I and type II $I - f$ curves.

Rebound spiking/bursting

The following paragraphs show an example of "rebound activity" with the eLIF model (S2 Fig), as well as details about the conditions leading to rebound activity for the AdExp and mAdExp models.

AdExp For the AdExp model, rebound spiking occurs [3] either:

- for type I excitability ($a/g_L < \tau_m/\tau_w$)
  - a) if $\tau_m/\tau_w < 1$
  - or b) if $\frac{\tau_m}{4\tau_w}\left(1 - \frac{\tau_w}{\tau_m}\right)^2 < a/g_L$
- in all situations for type II excitability ($a/g_L > \tau_m/\tau_w$)
  - a) if $\tau_m/\tau_w < 1$
  - or b) if $\tau_m/\tau_w \geq 1$

Cases I.b and II.b correspond to a neuron exhibiting dampened oscillations, so the presence of the sag is obvious. For cases I.a and II.a, the faster timescale associated to the membrane potential conditions the presence of a sag. Because type II excitability with $\frac{\tau_m}{4\tau_w}\left(1 - \frac{\tau_w}{\tau_m}\right)^2 > a/g_L$ is impossible, as $\frac{\tau_m}{4\tau_w}\left(1 - \frac{\tau_w}{\tau_m}\right)^2 <$

$\frac{\tau_m}{\tau_w} < \frac{a}{g_L}$ for $\tau_m/\tau_w \geq 0$, this covers all cases. Thus, rebound spiking in the AdExp model is always associated to a sag.

This can also be shown mathematically for I.a and II.a by looking at the eigenvector associated to the lowest eigenvalue:

$$\lambda_- = -\frac{\tau_m}{2\tau_w}\left[1 + \frac{\tau_w}{\tau_m} + \sqrt{\left(1-\frac{\tau_w}{\tau_m}\right)^2 - 4\frac{a\tau_w}{g_L\tau_m}}\right] \quad \text{and} \quad \mathbf{e}_- = \begin{pmatrix} \frac{2\tau_w/\tau_m}{1-\frac{\tau_w}{\tau_m}+\sqrt{\left(1-\frac{\tau_w}{\tau_m}\right)^2-4\frac{a\tau_w}{g_L\tau_m}}} \\ 1 \end{pmatrix} \qquad \text{(S9 Eq)}$$

For $\tau_m/\tau_w < 1$ (I.a and II.a), the denominator d of the x component of $\mathbf{e}_-$ gives its sign, and since

$$d \;=\; 1 - \frac{\tau_w}{\tau_m} + \sqrt{\left(1-\frac{\tau_w}{\tau_m}\right)^2 - 4\frac{a\tau_w}{g_L\tau_m}} \;<\; 1 - \frac{\tau_w}{\tau_m} + \left|1-\frac{\tau_w}{\tau_m}\right| \;=\; 0 \qquad \text{(S10 Eq)}$$

one can see that, as expected from the ratio of timescales, there is always an overshoot and a sag for I.a and II.a.

mAdExp The new rebound bursting behavior is associated to a positive divergence of the V-nullcline (cf. S7 Eq). Since the divergence occurs for

$$V^* = V_{th} - \Delta_T \ln\left(\frac{E_u - E_0}{\Delta_T}\right), \qquad \text{(S11 Eq)}$$

the positive sign is obtained for

$$V_{th} \leq E_u - \Delta_T \ln\left(\frac{E_u - E_0}{\Delta_T}\right) - \frac{\epsilon_c}{\epsilon_0}(E_u - E_L) + \frac{I_e - w}{g_L}. \qquad \text{(S12 Eq)}$$

To get the mAdExp model to display rebound spiking and no sag one must combine the previous condition with the constraints of the AdExp and eLIF models:

- the condition for no overshoot is a type I neuron with either $\frac{\tau_m}{\tau_w} > 1$ or $\frac{a}{g_L} > \frac{\tau_m}{4\tau_w}\left(1 - \frac{\tau_w}{\tau_m}\right)^2$, or any neuronal type with $a \leq 0$ (note that for small values of a, the sag, though technically present, can be neglected for all practical purposes),
- the condition for no overshoot due to energy dynamics is $E_u \geq E_0$ (necessary for the).

E. Parameters

Detailed parameter sets used in the different figures can be found in the following tables.

	Fig 3		Fig 4		S1 Fig		S2 Fig	
	**Value**	**Unit**	**Value**	**Unit**	**Value**	**Unit**	**Value**	**Unit**
C_m	100.	pF	200.	pF	100.	pF	100.	pF
g_L	9.	nS	12.	nS	9.	nS	9.	nS
E_0	-62.5	mV	-58.5	mV	$-69.$	mV	$-61.$	mV
I_e	0.	pA	35.	pA	0.	pA	0.	pA
E_u	-58.5	mV	$-55.$	mV	$-62.$	mV	$-65.$	mV
V_{th}	$-60.$	mV	$-53.$	mV	$[-65.5, -59.]$	mV	-60.5	mV
α	1.		1.		1.		1.	
E_d	$-40.$	mV	0.	mV	0.	mV	$-40.$	mV
E_f	$-62.$	mV	$-55.$	mV	$-66.$	mV	$-62.$	mV
ϵ_0	0.5		0.5		0.5		0.5	
ϵ_c	0.18		0.15		0.1		0.2	
δ	0.018		0.02		$\{0, 0.01\}$		0.02	
V_{reset}	$-62.$	mV	$-57.$	mV	$-66.$	mV	$-62.$	mV
t_{ref}	0.	ms	2.	ms	2.	ms	2.	ms
τ_e	200.	ms	500.	ms	1000.	ms	200.	ms

S2 Table. Parameters used with the eLIF model.

E.1. Neuronal parameters

	**RS**	**AS**	**IB**	**RB**	**TS**	**DB**	**DA**	**IR**	**ER**	**IS**	**Unit**
C_m	104	104	130	130	100	100	84	40	104	84	pF
g_L	4.3	4.3	18	8	9	6	5	6	4.4	5	nS
E_0	-64	-52.5	-56	-55	-56	-62.5	-52.5	-59.6	-54.4	-52.5	mV
V_{th}	-58	-52	-53	-54	-52	-55	-52	-58	-55	-52	mV
Δ_T	0.8	0.8	2	2	1.2	1.2	0.8	2	0.9	0.8	mV
a	0	2	2	3	51	-0.1	-0.5	1	0	-0.5	pA
τ_w	20	300	150	110	300	20	150	200	150	150	ms
b	0.5	5	50	60	150	35	0	20	5	0	pA
V_{reset}	-61	-54	-52.5	-50	-50	-53	-56	-58	-58	-54	mV
t_{ref}	0	0	0	0	0	0	0	0	0	0	ms
E_u	-60	-45	-52	-50	-52	-60	-45	-59	-51	-45	mV
α	1	1	1	1	1	1	1	1.5	1	0.5	
E_d	-40	-35	-20	-35	-30	-20	-35	-35	0	-20	mV
E_f	-46	-45	-45	-45	-45	-45	-45	-60	-35	-35	mV
ϵ_0	0.5	0.5	0.5	0.5	0.5	5	5	5	5	2	
ϵ_c	0.15	0.15	0.15	0.15	0.15	1.5	1	2	2	0.3	
δ	0.02	0.02	0.02	0.02	0.02	0.1	0.4	0.2	0.5	0.15	
γ	1000	200	200	300	200	500	200	500	200	200	pA
τ_e	500	500	500	150	500	50	200	100	500	2000	ms
I_{KATP}	1	1	1	1	1	100	100	5	1	100	pA
I_{low}	50	50	100	100	85	57	40	-36	30	10	pA
I_{high}	300	200	250	300	400	300	100	200	100	250	pA

S3 Table. Parameters used for the different behaviors of the mAdExp model on Fig 6.

	**Rebound (no sag)**		**Rebound (no sag)**		
	mAdExp	AdExp	mAdExp	AdExp	Unit
C_m	80	62.5	50.	47.	pF
g_L	3.2	2.5	2.	1.9	nS
E_0	-61.7	/	-60.	/	mV
E_L	/	-62.1	/	-71.	mV
V_{th}	-54.1	-54.3	-57.5	-56.2	mV
Δ_T	3.9	3.	3.	3.	mV
a	0.2	1.	1.8	1.4	pA
τ_w	500.	500.	250.	320.	ms
b	1.5	5.	10.	5.7	pA
V_{reset}	-56.5	-56.5	-53.	-53.	mV
t_{ref}	2.	2.	2.	2.	ms
I_e	0.	0.	0.	0.	ms
E_u	-61.5	/	-48.	/	mV
α	1.8	/	1.	/	
E_d	-26.	/	0.	/	mV
E_f	-65.	/	-40.	/	mV
ϵ_0	10.	/	10.	/	
ϵ_c	1.	/	8.	/	
δ	0.2	/	4.	/	
γ	1000.	/	200	/	pA
τ_e	15.	/	7.	/	ms
I_{KATP}	0.1	/	0.1	/	pA

S4 Table. Parameters used to match rebound spiking behaviors on Fig 7.

E.2. Network simulation

These are the parameters used for the network simulations of Fig 5.

	Value	Unit
C_m	200	pF
g_L	12	nS
E_0	-58.5	mV
I_e 0	pA	
E_u	-57	mV
E_d	0	mV
E_f	-48	mV
ϵ_0	0.5	
ϵ_c	0.15	
δ	0.007	
t_{ref}	2	ms
τ_e	500	ms

α	V_{th} (mV)	V_{reset} (mV)	s_e (pA)
1	-55.3	-60	30
0.6	-55.3	-57	4.41
0.3	-54.66	-57	2.91
0.2	-54.44	-57	2.7

S5 Table. Left: static neuronal parameters used with the all eLIF neurons in Fig 5. Right: Specific parameters used for each of the simulations at a different neuronal health in Fig 5. Each health level, corresponding to a value of α is associated to the corresponding values for V_{th} and V_{reset} in the same column.

The networks were all Erdös–Renyi graphs containing 800 excitatory neurons and 200 inhibitory neurons, each having an average degree of 100. The networks were generated using the NNGT library [4]. Even for the healthy situation with $\alpha = 1$, the network was placed in the inhibition-dominated regime, with an inhibitory synaptic strength 120 pA and a synaptic timescale of 2 ms against excitatory synapses with strength 30 pA and a timescale of 0.2 ms. The inhibitory strength and all timescales were kept unchanged for all conditions, only excitatory strength was scaled according to the values s_e in S5 Table.

References

1. Fardet T, Vennemo SB, Mitchell J, Mørk H, Graber S, Hahne J, et al.. NEST 2.20.0; 2020. Available from: `https://doi.org/10.5281/zenodo.3605514`.

2. Nickalls RWD. A New Approach to Solving the Cubic: Cardan's Solution Revealed. The Mathematical Gazette. 1993;77(480):354. doi:10/bq9w7j.

3. Touboul J, Brette R. Dynamics and Bifurcations of the Adaptive Exponential Integrate-and-Fire Model. Biological Cybernetics. 2008;99(4-5):319–334. doi:10/c3ww2z.

4. Fardet T. NNGT 2.0: full frontend for simple graphs, unified graph analysis; 2020. Available from: `https://doi.org/10.5281/zenodo.3903176`.

List of Figures

S1 Fig $I - f$ curves of the eLIF neuron for different threshold values V_{th} (left/right). The corresponding phase-space is shown in the middle. Threshold values are -65.5 (dark grey), -63, -61, and -59 mV (light grey); they correspond to the associated curves on the $I - f$ plots and to the dashed vertical lines on the phase-space representation. The type of the curve depends on the position of V_{th} compared to the position of the low-energy fixed point (FP) at the bifurcation point which is shown as a filled black circle: for $V_{th} > V_{FP}$, the neuron has a continuous type I response curve whereas for $V_{th} > V_{FP}$ the curve, though still continuous, becomes closer to a type II curve, with a sharp increase starting immediately at the bifurcation current I_e^*. See S2 Table for detailed parameters.

S2 Fig Dynamics of the eLIF model as timeseries (A) and in phase-space (B) for $E_u < E_0$ (resonant behavior). The behavior of the model is shown in response to four different inputs, shown in grey on the V subplot: a low depolarizing current (a: 10 pA), a stronger depolarizing current (b: 30 pA), a large depolarization (c: 80 pA), and a hyperpolarizing current (d: -60 pA). Corresponding behavior in phase-space is shown in the four right panels, with spike emission marked by an empty left triangle and reset position marked by a dot: (a) the neuron leaves the fixed point (FP), then goes back towards it (both transitions are associated to and up/downshoot), (b) the neuron spikes at decreasing frequency as its energy is depleted, (c) the neuron spikes, then enters a depolarization block for high stimulation, (d) post-inhibitory overshoot is associated to rebound spiking. See S2 Table for detailed parameters.

List of Tables

S1 Table Runtime of various models in NEST. A "baseline" run with no neuron (None), compared to runs with one neuron of each of the mentioned models. For the new energy-based models (eLIF and mAdExp), two runs were performed: one using a naive implementation and another using slightly optimized implementation (numbers in parentheses). Conductance-based models are also included: a standard Hodgkin-Huxley (HH) model which can display regular spiking an depolarization block, and one with calcium and calcium-gated potassium (HH+Ca) to reproduce bursting dynamics 2

S2 Table Parameters used with the eLIF model. 6

S3 Table Parameters used for the different behaviors of the mAdExp model on Fig 6. 6

S4 Table Parameters used to match rebound spiking behaviors on Fig 7. 7

S5 Table Left: static neuronal parameters used with the all eLIF neurons in Fig 5. Right: Specific parameters used for each of the simulations at a different neuronal health in Fig 5. Each health level, corresponding to a value of α is associated to the corresponding values for V_{th} and V_{reset} in the same column. 8
