# Supplementary material for: Simple models including energy and spike constraints reproduce complex activity patterns and metabolic disruptions
Source: PLoS Comput Biol. 2020 Dec 21;16(12):e1008503. doi: 10.1371/journal.pcbi.1008503 (PMC7785241; doi:10.1371/journal.pcbi.1008503)
Supplement: S1 Table — A “baseline” run with no neuron (None), compared to runs with one neuron of each of the mentioned models. For the new energy-based models (eLIF and mAdExp), two runs were performed: one using a naive implementation and another using slightly optimized implementation (numbers in parentheses). Conductance-based models are also included: a standard Hodgkin-Huxley (HH) model which can display regular spiking an depolarization block, and one with calcium and calcium-gated potassium (HH+Ca) to reproduce bursting dynamics. (PDF) [file pcbi.1008503.s004.pdf]

**Model**	None	LIF	AdExp	*e*LIF	mAdExp	HH	HH+Ca
**Runtime (s)**	0.75	0.8	2.7	2.86 (1.79)	3.52 (2.56)	3.47	4.92

S1 Table. Runtime of various models in NEST. A “baseline” run with no neuron (None), compared to runs with one neuron of each of the mentioned models. For the new energy-based models (*e*LIF and mAdExp), two runs were performed: one using a naive implementation and another using slightly optimized implementation (numbers in parentheses). Conductance-based models are also included: a standard Hodgkin-Huxley (HH) model which can display regular spiking an depolarization block, and one with calcium and calcium-gated potassium (HH+Ca) to reproduce bursting dynamics
