# Supplementary material for: Simple models including energy and spike constraints reproduce complex activity patterns and metabolic disruptions
Source: PLoS Comput Biol. 2020 Dec 21;16(12):e1008503. doi: 10.1371/journal.pcbi.1008503 (PMC7785241; doi:10.1371/journal.pcbi.1008503)
Supplement: S2 Table — (PDF) [file pcbi.1008503.s005.pdf]

	Fig 3		Fig 4		S1 Fig		S2 Fig	
	**Value**	**Unit**	**Value**	**Unit**	**Value**	**Unit**	**Value**	**Unit**
C_m	100.	pF	200.	pF	100.	pF	100.	pF
g_L	9.	nS	12.	nS	9.	nS	9.	nS
E_0	-62.5	mV	-58.5	mV	$-69.$	mV	$-61.$	mV
I_e	0.	pA	35.	pA	0.	pA	0.	pA
E_u	-58.5	mV	$-55.$	mV	$-62.$	mV	$-65.$	mV
V_{th}	$-60.$	mV	$-53.$	mV	$[-65.5, -59.]$	mV	-60.5	mV
α	1.		1.		1.		1.	
E_d	$-40.$	mV	0.	mV	0.	mV	$-40.$	mV
E_f	$-62.$	mV	$-55.$	mV	$-66.$	mV	$-62.$	mV
ϵ_0	0.5		0.5		0.5		0.5	
ϵ_c	0.18		0.15		0.1		0.2	
δ	0.018		0.02		$\{0, 0.01\}$		0.02	
V_{reset}	$-62.$	mV	$-57.$	mV	$-66.$	mV	$-62.$	mV
t_{ref}	0.	ms	2.	ms	2.	ms	2.	ms
τ_e	200.	ms	500.	ms	1000.	ms	200.	ms

S2 Table. Parameters used with the *e*LIF model.
