# Supplementary material for: Simple models including energy and spike constraints reproduce complex activity patterns and metabolic disruptions
Source: PLoS Comput Biol. 2020 Dec 21;16(12):e1008503. doi: 10.1371/journal.pcbi.1008503 (PMC7785241; doi:10.1371/journal.pcbi.1008503)
Supplement: S3 Table — (PDF) [file pcbi.1008503.s006.pdf]

	**RS**	**AS**	**IB**	**RB**	**TS**	**DB**	**DA**	**IR**	**ER**	**IS**	**Unit**
C_m	104	104	130	130	100	100	84	40	104	84	pF
g_L	4.3	4.3	18	8	9	6	5	6	4.4	5	nS
E_0	-64	-52.5	-56	-55	-56	-62.5	-52.5	-59.6	-54.4	-52.5	mV
V_{th}	-58	-52	-53	-54	-52	-55	-52	-58	-55	-52	mV
Δ_T	0.8	0.8	2	2	1.2	1.2	0.8	2	0.9	0.8	mV
a	0	2	2	3	51	-0.1	-0.5	1	0	-0.5	pA
τ_w	20	300	150	110	300	20	150	200	150	150	ms
b	0.5	5	50	60	150	35	0	20	5	0	pA
V_{reset}	-61	-54	-52.5	-50	-50	-53	-56	-58	-58	-54	mV
t_{ref}	0	0	0	0	0	0	0	0	0	0	ms
E_u	-60	-45	-52	-50	-52	-60	-45	-59	-51	-45	mV
α	1	1	1	1	1	1	1	1.5	1	0.5	
E_d	-40	-35	-20	-35	-30	-20	-35	-35	0	-20	mV
E_f	-46	-45	-45	-45	-45	-45	-45	-60	-35	-35	mV
ϵ_0	0.5	0.5	0.5	0.5	0.5	5	5	5	5	2	
ϵ_c	0.15	0.15	0.15	0.15	0.15	1.5	1	2	2	0.3	
δ	0.02	0.02	0.02	0.02	0.02	0.1	0.4	0.2	0.5	0.15	
γ	1000	200	200	300	200	500	200	500	200	200	pA
τ_e	500	500	500	150	500	50	200	100	500	2000	ms
I_{KATP}	1	1	1	1	1	100	100	5	1	100	pA
I_{low}	50	50	100	100	85	57	40	-36	30	10	pA
I_{high}	300	200	250	300	400	300	100	200	100	250	pA

S3 Table. Parameters used for the different behaviors of the mAdExp model on Fig 6.
