# Supplementary material for: Simple models including energy and spike constraints reproduce complex activity patterns and metabolic disruptions
Source: PLoS Comput Biol. 2020 Dec 21;16(12):e1008503. doi: 10.1371/journal.pcbi.1008503 (PMC7785241; doi:10.1371/journal.pcbi.1008503)
Supplement: S4 Table — (PDF) [file pcbi.1008503.s007.pdf]

	**Rebound (no sag)**		**Rebound (no sag)**		
	mAdExp	AdExp	mAdExp	AdExp	Unit
C_m	80	62.5	50.	47.	pF
g_L	3.2	2.5	2.	1.9	nS
E_0	-61.7	/	-60.	/	mV
E_L	/	-62.1	/	-71.	mV
V_{th}	-54.1	-54.3	-57.5	-56.2	mV
Δ_T	3.9	3.	3.	3.	mV
a	0.2	1.	1.8	1.4	pA
τ_w	500.	500.	250.	320.	ms
b	1.5	5.	10.	5.7	pA
V_{reset}	-56.5	-56.5	-53.	-53.	mV
t_{ref}	2.	2.	2.	2.	ms
I_e	0.	0.	0.	0.	ms
E_u	-61.5	/	-48.	/	mV
α	1.8	/	1.	/	
E_d	-26.	/	0.	/	mV
E_f	-65.	/	-40.	/	mV
ϵ_0	10.	/	10.	/	
ϵ_c	1.	/	8.	/	
δ	0.2	/	4.	/	
γ	1000.	/	200	/	pA
τ_e	15.	/	7.	/	ms
I_{KATP}	0.1	/	0.1	/	pA

S4 Table. Parameters used to match rebound spiking behaviors on Fig 7.
