# Supplementary material for: Simple models including energy and spike constraints reproduce complex activity patterns and metabolic disruptions
Source: PLoS Comput Biol. 2020 Dec 21;16(12):e1008503. doi: 10.1371/journal.pcbi.1008503 (PMC7785241; doi:10.1371/journal.pcbi.1008503)
Supplement: S5 Table — Left: static neuronal parameters used with the all eLIF neurons in Fig 5. Right: Specific parameters used for each of the simulations at a different neuronal health in Fig 5. Each health level, corresponding to a value of α is associated to the corresponding values for Vth and Vreset in the same column. (PDF) [file pcbi.1008503.s008.pdf]

	Value	Unit
C_m	200	pF
g_L	12	nS
E_0	-58.5	mV
I_e 0	pA	
E_u	-57	mV
E_d	0	mV
E_f	-48	mV
ϵ_0	0.5	
ϵ_c	0.15	
δ	0.007	
t_{ref}	2	ms
τ_e	500	ms

α	V_{th} (mV)	V_{reset} (mV)	s_e (pA)
1	-55.3	-60	30
0.6	-55.3	-57	4.41
0.3	-54.66	-57	2.91
0.2	-54.44	-57	2.7

S5 Table. Left: static neuronal parameters used with the all *e*LIF neurons in Fig 5. Right: Specific parameters used for each of the simulations at a different neuronal health in Fig 5. Each health level, corresponding to a value of α is associated to the corresponding values for V_{th} and V_{reset} in the same column.
