# Supplementary material for: Simple models including energy and spike constraints reproduce complex activity patterns and metabolic disruptions
Source: PLoS Comput Biol. 2020 Dec 21;16(12):e1008503. doi: 10.1371/journal.pcbi.1008503 (PMC7785241; doi:10.1371/journal.pcbi.1008503)
Supplement: Attachment — Submitted filename: response_to_reviewers.pdf [file pcbi.1008503.s009.pdf]

Responses to reviewers

Reviewer 1 Response

> The authors claim (first sentence of Abstract) to introduce new models that “account for energy supply and demand”. In reality, they reproduce phenomena like depolarization block and bistability/ bursting by introducing a slow variable whose connection to energy is not so clear. These phenomena have been previously shown to emerge from, amongst other things, changes in ion reversal potentials that occur if/when ion flux is tracked AND when the resulting ion concentration changes are not counteracted by pumps or co-transporters that (directly or indirectly) rely on ATP consumption. The dynamics introduced by slow ion concentration changes have actually been the subject of significant modeling, which is not adequately discussed in this manuscript. Those past papers may not have referred to the slow variable as “energy”, but there is certainly a connection. The novelty of the current paper is much reduced once one recognizes this connect.

In previous models, slow variables were usually introduced to model adaptation using calcium-gated potassium and bursting dynamics. Conversely, the eLIF and mAdExp models provide the epsilon variable as a way to explicitly model energy-related and spike-initiation constraints. This significant difference in interpretation allows designing straightforward models for energy-related disorders. At the same time, it makes it possible to combine energy with other slow variables (here, mAdExp). We demonstrated how some behaviors even in the healthy cells can be reproduced only with the help of both energy and adaptation variables (Fig. 7). Under metabolic stress, the clear separation between the variables becomes even more essential.

We have developed the "Discussion" sections (4.1 and 4.2) to explain how the "energy variable" epsilon that we introduced differs from the previously studied slow variables. Apart from the fact that they usually evolve with timescales that differ from the membrane timescale, they have little in common, especially when it comes to how they affect neuronal excitability.

> Despite providing a reasonable introduction about the importance of energy for neuron function, the modeling failed to connect energy consumption and neuronal excitability in a meaningful way. According to equations, E_L is dependent epsilon (energy) but

> the relationship is not explained. Why is the generation of energy written the way it is? For the mAdExp model, w also depends on I_katp, but, here again, the relationship is not explained and one is left wondering if parameters have any biological meaning/constraint, or are simply adjusted to make the model work. On lines 312-313, the authors write that “epsilon cannot be quantitatively related to biological measurement”, but even the qualitative mapping falls short. Granted, the authors want to create a simple model amenable to analysis and large-scale simulations, but in justifying the models they propose, they might have created a more biophysically detailed model in which energy-dependent mechanisms are explicitly modeled, and then formally reduced it to the forms presented here… or they might have derived their equations from known relationships that they can point to in the literature.

Thank you, we realized that it was a major shortcoming and now corrected for it.

We have expanded the "Methods" sections (2, 2.1, and 2.2) to provide more details and improve clarity. We discussed explicitly the mapping between terms and parameters in the equations and associated biological mechanisms. In particular, we related the changes in the resting potential to the state of the Na/K pump (which depends on the energy level) and have discussed ATP-gated potassium channels. We also explicitly pointed out that the phenomenological behaviors we implemented were observed in previous studies using more detailed models.

> Without this, the modeling seems circular – models produce the “behavioral requirements” they were designed to reproduce – and one is left wondering if that is really because of energy, and if the models can do more than they were designed for.

It is worth noting that, though rebound spiking was a requirement for a successful model, we did not actively try to incorporate it via energy-related mechanisms since the AdExp neuron already display such behavior.
Therefore, the new mechanism enabling no-sag rebound is not a property that was mAdExp was *designed* to reproduce but an emergent feature that stemmed from interactions between adaptation and energy in the model.
See also the collective dynamics discussion below for more emergent properties.

> Indeed, the authors speculate about synchrony and information processing, but test neither; based on their mention of large-scale simulations in the Abstract, I was expecting more.

We initially did not want to shift the focus away from the main point: how energy can be

introduced in phenomenological integrate-and-fire models to obtain relevant and currently unavailable dynamics. Though these behaviors are indeed all the more relevant when considered within a collective dynamics, we felt that mixing the analysis of the single-neuron properties with network activity would make the paper lengthy and hard to read.

To not shift the main focus but still exemplify the use of the model in the recurrent network setting, we added a new part into "Transition from health to disease." There we illustrate with new simulations how a balanced network can react to a decrease in metabolic health. We found that, if homeostatic regulations are in place, the low input regime dynamics remains mostly unchanged for a broad range of alpha-parameter. However, if some neurons are injected with an additional external input, they progressively switch from continuous tonic firing (as in the healthy balanced network) to well-separated bursts (in the network with significantly decreased metabolic health, Figure 5). Clearly, this is just illustratory examinations, but we hope to inspire further investigations of energetic impact on recurrent activity.

Additionally, we checked the feasibility of large-scale simulations using our models. To this end, we benchmarked them against the most commonly used models (Appendix A.2). With small optimization, the full mAdExp model was faster than the standard implementation of the AdExp model that was used in other studies for large scale simulations on HPC hardware.

Reviewer 2 Response

> I have some objections regarding the “energy” aspect of the proposed models. The proposed models have a neural component and an energy component. For the former, the authors used standard spiking neuron models which have been proven to explain real neural firing patterns under certain conditions. That is, their link to real neural spiking was studied extensively. But in case of energy component of the proposed model, no effort seems to be made to link it to real energy substrates in the brain like ATP, pyruvate, lactate glucose etc. In the conceptual schematic of fig 1, the authors state that the energy ATP/ADP etc but it only remains a general comment. It does not reflect in the mathematical development of the equations. Here again, in eLIF model, the energy dynamics is completely abstract and the authors do not even comment on its relation to real metabolic substrates of energy in the brain. In mAdExp model, in the w-dynamic equation, there is a IKATP term where the model makes its first contact with

> its biophysical substratum. But, unfortunately, the authors don't define IKATP term and only make an unsubstantiated comment that I_KATP is used… "to account for ATP-sensitive potassium channels that trigger potassium outflow when the ATP/ADP ratio becomes small." The authors need to explain this comment in much greater detail and show how the proposed energy dynamics has a deeper biophysical grounding.

Thank you for pointing out the ambiguity in our formulations, this helped us revise the manuscript: We have largely expanded the "Methods" sections (2, 2.1, and 2.2) to provide more details and improve the clarity of the mapping between terms and parameters in the equations and associated biological mechanisms. In particular, we relate now the changes in the resting potential to the state of the Na/K pump (which depends on the energy level) and we have discussed ATP-gated potassium channels.

> In this respect, the authors must refer to the work of (Chhabria and Chakravarthy 2016). In this study, the authors describe spiking neuron models with an associated dynamics of an energy variable, analyse different dynamical regimes and link them to normal and energy-starved conditions. The model formulation in (Chhabria and Chakravarthy 2016) has a strong similarity to the proposed models in the current manuscript, which does not cite the previous study.
> The similarity is even more interesting if you consider that (Chhabria and Chakravarthy 2016) also used I_KATP current to make contact with real biophysical "energy." Therefore, the authors of the current must make a detailed comparison of their models with those mentioned above and justify the novelty, if any, of their proposed model.

We have discussed the work of Chhabria and Chakravarthy (2016) in the revised manuscript (4.1 and 4.2). Indeed, this work was unwarrantedly overlooked, it escaped our search due to its title and a slightly different focus. Though the models may look similar at first glance, the Model 2 presented in the Chhabria and Chakravarthy paper is mathematically much closer to a GLIF model than to any of our models, making the similarities between the models mostly related to the metabolic focus. One of the important difference points is that our model explicitly separates calcium-gated potassium adaptation and energetic constraints. We add to the paper a detailed discussion of the differences between our approach and the model of Chhabria and Chakravarthy.

> Minor comments:
>
> 1. In Fig. 3, in the subplots on the right side, in the subplot b, the voltage fluctuation seems to be confined roughly to

> $$-65mV, -60mV$$
>
> but in the corresponding voltage plot on the left top, voltage seems to fluctuate between roughly -60 mV and -50 mV. How do you explain this discrepancy? Same thing is true with subplot c.

On the left subplot, spike times have been marked by setting the voltage to -50 mV for visualization purposes. This is now mentioned in the caption.

> 2. In fig 4, where dynamics under energy-starved or pathological conditions are described, whether the phase plot has 3 FPs or 1 FP depends on the value of I_e also and not just alpha. So does the change in the input current Ie also represent pathology? Which doesn't make much sense. Can the authors explain this?

A similar transition is indeed possible by changing either alpha or I_e.

This is reasonable since from a single neuron perspective, increasing I_e or alpha would biologically be associated with increased internal sodium concentration and place the neuron in similar conditions. Extreme currents or deficient health would both eventually lead the neuron to enter a non-responsive state, then die. (E.g, it was observed that the high input current over a prolonged period of time brings neurons to the depolarization block.)

However, the critical difference between these two procedures is that changes in I_e biologically happen on very fast timescales (tens of milliseconds to a second) in clamping experiments and is reversible. In contrast, changes in alpha would occur over days, weeks, or months, and often be irreversible. At the same time, if the rise of the external input is chronic, it probably indicates a failure in some of the regulation mechanisms in the neuronal circuits.

> Typos:
> On line 219: “bifurcations” instead of “bifurcation”

The use of plural form was intended, there are multiple bifurcations that we were referring to.

> On line 234: “ends” instead of “end”
> On line 305: “shortcomings” instead of “shortcoming”

Thank you for spotting, these were corrected and the text was thoroughly proof-read.

Reviewer 3 Response

> The dynamics of neurons depends on many physiological parameters. The authors provide two phenomenological single-cell models that incorporate the effect of the metabolic supply – a variable that is particularly relevant in situations of stress and pathology.
>
> They equip the standard LIF model with energy dynamics, including contributions of the consumption arising from subthreshold membrane potential changes as well as action potentials on a phenomenological level. In addition, they also propose an adaptive version of an exponential integrate-and-fire model with an additional dimension and a representation of a sodium-potassium pump current. The authors argue that this approach captures a wide variety of spiking dynamics (including rebound spikes without inhibitory sag) and also takes into account the impact of the energetic supplies. In contrast to the usual phenomenological threshold models, these energetically extended models can also capture the dynamical state of depolarization block.
>
> The idea of combining simple phenomenological models with an energetic component is excellent and paves the road towards larger-scale network simulations and analyses that incorporate the metabolic state. Existing data across qualitatively different dynamics can be captured by the models (the authors provide examples) and the models also allow one to investigate the effects of (usually slow) changes in metabolic supply and the transitions between healthy (energy-rich) and pathological (energy-deprived) states.
>
> The manuscript is well written and the analyses are described clearly. I do not hesitate to give an immediate recommendation to accept the manuscript without major revision. I am convinced that it provides an interesting novel contribution that will meet the interest of the community. The authors may have considered to end their analysis with a network simulation, providing an example for the usefulness of the models in the larger network context. But I do not consider this a requirement.

Thank you for the supportive comments and suggestions!

Initially, we wanted to keep the focus of the paper on how the energy can be introduced in phenomenological integrate-and-fire models to obtain relevant and currently unavailable dynamics. We feared that adding the network aspect would make the paper lengthy and

hard to read.

However, after additional consideration, we decided to exemplify the use of the model in the recurrent network setting. We added a new part into "Transition from health to disease", where we illustrated with new simulations how the balanced network reacts to the decrease in metabolic health. We found that if homeostatic regulations are in place, the dynamics in the low input regime can remain largely unchanged for a broad range of alpha-parameter. However, if some neurons are injected with an additional external input, they progressively switch from continuous tonic firing (as in the healthy balanced networks) to well-separated bursts (in the network with significantly decreased metabolic health), see Figure 5. Clearly, this is just illustratory examinations, but we hope that they will inspire further investigations of energetic impact on recurrent activity.
