# Supplementary material for: Simple models including energy and spike constraints reproduce complex activity patterns and metabolic disruptions
Source: PLoS Comput Biol. 2020 Dec 21;16(12):e1008503. doi: 10.1371/journal.pcbi.1008503 (PMC7785241; doi:10.1371/journal.pcbi.1008503)
Supplement: Attachment — Submitted filename: resp_round2.pdf [file pcbi.1008503.s010.pdf]

Responses to reviewers

> 1. The authors responded to my first point that “slow variables were usually introduced to model adaptation using calcium-gated potassium and bursting dynamics”. But there are a lot more ions to consider that just calcium, and (relatively slow) changes in those ion concentrations have been show to introduce slow dynamics into neuron models (directly by changing reversal potentials, which in turn alters the strength of different currents, amongst other effects). A quick Pubmed search turns up dozens of such models. I list just a few to make sure the authors understand what I am referring to:
>   https://pubmed.ncbi.nlm.nih.gov/30180647/
>   https://pubmed.ncbi.nlm.nih.gov/22058273/
>   https://pubmed.ncbi.nlm.nih.gov/10899222/
>   I think my original point was pretty clear. I agree that adaptation has been extensively modeled as a calcium-dependent process, but the authors need to consider other slow processes that impact neuronal spiking in ways directly relevant for this study.

We thank the reviewer for continuing the discussion, we believe that it allowed clarifying the motivation behind the model and choices we made in its design.

In brief: The main goal of our phenomenological models is to provide a way to study how metabolic constraints can also contribute to or modulate network dynamics. If other mechanisms are deemed necessary in later studies, researchers can include any additional slow (or not) mechanisms in the model and study their interactions with metabolic constraints.

Detailed answer: All three papers deal with (sometimes multicompartmental) conductance-based models, and reference 2 (Coggan et al. 2003) also provides a Morris-Lecar-like simplification of the initial multicompartmental model.
We may not have put enough emphasis on this in the discussion and introduction, but we explicitly excluded conductance-based models (and especially multi-compartmental versions) because their computational cost is too high to allow large-scale simulations and make the dynamical analysis complex, and even usually completely untractable analytically, except for very specific features.
This is one of the reasons why studies using such models always use extremely small-sized

networks, on the order of 100 neurons.

We, therefore, consider that references 1 and 3 are simply other examples of conductance-based models that complement the studies already cited in our paper [8,9,25,26].

The 3D Morris-Lecar neuron in Coggar et al. (2003), like the other models in references 1 and 3, uses slow dynamics to describe one potential mechanism for depolarization blocks and spreading depression. It uses persistent sodium channels to create bistability between repetitive spiking and depolarization block. The most significant similarity between the two models is that they share the same analysis on the causes of depolarization block (i.e. the same phenomenology for the mechanism: internal sodium accumulation).
Thus, our two models investigate different contributions to a single behavior: while Coggan et al focus on one potential mechanism for the depolarization block, our model provides, among many other features, a way to investigate how metabolic constraints can enhance or limit sodium accumulation inside the neuron and thus modulate the depolarization block.

We do not think that discussing all non-metabolic mechanisms that can lead to depolarization block through other (necessarily slow) dynamics would improve this study. Similarly, Coggar et al. do not discuss chloride and cell-volume because they are not relevant to their study. All of these mechanisms can necessarily occur in neurons and may contribute to the depolarization block. It is not the purpose of our paper to reproduce or discuss them, but simply to provide a way to study how metabolic constraints can also contribute to or modulate such dynamics.

We expanded corresponding discussions in the paper.

> 2. The authors have revised the text in the Methods section to help connect energy consumption and neuronal excitability, but the outcome fell short. On line 151-153, the authors explain that since “there is no simple relationship between membrane potential and energy consumption, we decided to use a 3rd order polynomial to give a nonlinear shape to the epsilon-nullcline.” Sorry, but I really don’t think that is a legitimate explanation. Why does the nullcline have that shape? The next sentence (because “this enables both quasi-linear behaviors and more complex dynamics”) is circular – we do it because it works. I am still trying to figure out a sigmoidal relationship between ATP/ADP ratio and voltage would arise.

The scope of this article is to provide low-dimensional integrate-and-fire models that enable modeling of energetic constraints on both fast and slow timescales, especially for applications related to neuronal disorders (see Figure 4), eventually making it possible to

simulate large-scale networks during the whole progression of the disease.
Since complex dynamics such as bursting, rebound spiking, and depolarization block are also highly relevant for the study of neuronal disorders, we made sure that our models were also able to reproduce these features. To achieve the ambitious objective of reproducing such a broad set of activities with a low-dimensional and analytically tractable model, we had to rely on some phenomenological mechanisms.

Since there is no available information about the functional form of the relationship between membrane potential and energy consumption, we have freedom in selecting the functional class. Our rationale is that we want on one side, to select the function allowing for a wide range of behaviors and relationships observed in experiments and on the other side be as simple as possible. We chose a 3rd order polynomial, the simplest nonlinearity that can, depending on parameter values, enable either 1) a behavior that is qualitatively equivalent to a linear relationship or 2) the presence of a bistability. The model will thus let us explore the consequences of the presence or absence of bistability.

We modified the discussion of the model-choice in the text, to highlight the reasoning behind the particular choice and a degree of arbitrariness that remains.

> There are other statements, which might seem minor, but raise concerns. For example, the authors suggest that sodium-channel inactivation occurs because “the Na/K pump cannot move sodium out quickly enough” and cite reference 28. Bikson et al. (2003) does not address intracellular sodium concentration; instead, I would interpret their data, which shows dramatic extracellular potassium accumulation in the context of seizure activity, as reducing post-spike repolarization which impairs recovery of Na channels from inactivation. Intracellular accumulation of sodium will reduce the Na reversal potential, and could contribute to depolarization that encourages Na channel inactivation, but intracellular sodium does not directly cause Na channel to inactivate. Such details may not be critical, but the onus is on the authors to argue that, which starts with an accurate description of the biological mechanism followed by a rational simplification for the purposes of modeling. For the record, I do not think that the model has to capture all of the biophysical detail, but I am disturbed when the authors rationalize their model based on misunderstood mechanisms.

The paper from Bikson et al. (2003) states in the abstract "Application of phenytoin or veratridine indicated a critical role for the persistent sodium current in maintaining depolarization block.", then in the discussion "Since no relationship was found between $[K_o^+]$ measured in stratum pyramidale and population spike disruption, other factors must

also contribute to the generation of the larger membrane depolarization (depolarization block) observed during intermittent-spiking events. Our results suggest that activation of persistent inward currents promoted the generation of depolarization block". Though this does indeed not state that intracellular sodium concentration causes sodium-channel inactivation, we do not think that our interpretation goes against the results of this paper.

Furthermore, in the standard HH model, the equation for h (the inactivation variable for Na) goes as:

$$h = \left[1 + \exp\left(\frac{V - V_h}{k_h}\right)\right]^{-1}$$

Since V depends mostly on variations of K_o^+ and Na_i^+, we do not see any issue with our interpretation, especially since, as the NKP moves sodium and potassium in opposite directions, reasoning on K_o^+ or Na_i^+ is often almost equivalent.

Finally, we would also like to point out that the 2nd reference provided by Reviewer 1, regarding models with slow variables, also happens to use slow intracellular sodium accumulation to explain depolarization block: "Repetitive spiking stops when Na+ accumulation brings the system to the end of the stable limit cycle branch."

> 3. I appreciate the addition of Figure 5. The legend text refers to red circles and gray triangles, which are not clear in the figure version embedded in the text, but do show up in the larger version of the figure. But in that larger version, shading is black rather than gray. The text says the input rate is increased between 1500 and 1600 ms, but the shaded region (and altered response) seems to extend from before 1400 to after 1600 ms.

We thank the reviewer for pointing this out. This was an issue with the EPS format and transparency. The correct figure with both symbols and grey shading visible can be seen by zooming on the embedded figure. We changed the figure to use a non-transparent shading in order to avoid this issue. We thank the reviewer also for noticing the error in the caption which was for an older version; we changed the figure and numbers to 1400 and 1650 ms to have "rounder" numbers.
